# Myelin damage and cortical atrophy in watershed regions in patients with moyamoya angiopathy

**DOI:** 10.3389/fnins.2022.982829

**Published:** 2022-08-23

**Authors:** Elena Filimonova, Konstantin Ovsiannikov, Alexsey Sosnov, Artem Perfilyev, Rustam Gafurov, Dmitriy Galaktionov, Anatoliy Bervickiy, Vitaly Kiselev, Jamil Rzaev

**Affiliations:** ^1^Federal Center of Neurosurgery, Novosibirsk, Russia; ^2^Department of Neurosurgery, Novosibirsk State Medical University, Novosibirsk, Russia; ^3^Department of Neuroscience, Institute of Medicine and Psychology, Novosibirsk State University, Novosibirsk, Russia

**Keywords:** moyamoya angiopathy (MMA), magnetic resonance imaging (MRI), T1w/T2w ratio, demyelination, MR-morphometry, FreeSurfer

## Abstract

**Background:**

Despite it being known that chronic ischemia results in myelin damage and gray matter atrophy, data regarding patients with moyamoya angiopathy is limited. We hypothesized that chronic ischemia in moyamoya angiopathy leads to myelin damage, especially in anterior watershed regions, as well as cortical atrophy in these areas.

**Materials and methods:**

Twenty adult patients with moyamoya angiopathy and 17 age- and sex-matched healthy controls were evaluated using the T1w/T2w mapping method and surface-based MR-morphometry. The T1w/T2w signal intensity ratio, which reflects the white matter integrity, and the cortical thickness, were calculated in watershed regions and compared between the patients and controls. In the patients with moyamoya angiopathy, the correlations between these parameters and the Suzuki stage were also evaluated.

**Results:**

The regional T1w/T2w ratio values from centrum semiovale in patients with MMA were significantly lower than those in healthy controls (*p* < 0.05); there was also a downward trend in T1w/T2w ratio values from middle frontal gyrus white matter in patients compared with the controls (*p* < 0.1). The cortical thickness of the middle frontal gyrus was significantly lower in patients than in healthy controls (*p* < 0.05). There were negative correlations between the Suzuki stage and the T1w/T2w ratio values from the centrum semiovale and middle frontal white matter.

**Conclusion:**

T1w/T2w mapping revealed that myelin damage exists in watershed regions in patients with moyamoya angiopathy, in association with cortical atrophy according to MR-morphometry. These changes were correlated with the disease stage.

## Introduction

Moyamoya angiopathy (MMA) is a rare cerebral vasculopathy described by steno-occlusion of the circle of Willis arteries, with the development of collateral vessels network (moyamoya vessels). In moyamoya disease, MMA is characterized by progressive steno-occlusion of the terminal segments of internal carotid arteries and/or their proximal branches as a single manifestation; whereas in moyamoya syndrome, MMA is associated with an underlying disease [Guidelines for Diagnosis and Treatment of Moyamoya Disease, 2012]. These changes result in chronic ischemia of the brain parenchyma with subsequent serious cerebrovascular accidents, especially strokes (Liu et al., 2020).

Watershed regions are the most prone to chronic ischemic changes (Mangla et al., 2011), especially in the case of steno-occlusive processes, such as atherosclerotic disease and MMA. It happens because that perfusion delay is stronger in these areas. In the case of moyamoya angiopathy the anterior external border zone regions (such as the middle frontal cortex, paramedian white matter, and centrum semiovale) are more susceptible to ischemia (Canavero et al., 2021).

It is believed, that chronic ischemia results in microstructural changes in the brain parenchyma (both white and gray matter) which appeared before the cerebrovascular accidents. Chronic ischemia is known to damage the structure of myelin, which has been shown in a few previous studies, particularly for MMA patients. Different methods, such as diffusion tensor imaging (Liu et al., 2020) and magnetization transfer saturation imaging (Hara et al., 2020) have been used to assess the myelin integration in this disorder and revealed the myelin damage in several brain regions. However, there is no consensus in this research area because of data heterogeneity, small sample sizes, etc. Additionally, all of these methods are difficult to use in routine clinical practice due to technical issues (sophisticated image acquisitions and/or long scanning time).

Another neuroimaging method of measuring the myelin integrity of the brain is the calculation of the T1w to T2w signal intensity ratio [or T1w/T2w mapping, Glasser and van Essen (2011)]. The signal intensity from white matter on T1-weighted images reflects the spatial distribution of myelin-bound cholesterol; conversely, the signal intensity from white matter on T2-weighted images relates to proton transfers, molecular exchange, and diffusion of water, and T2w-hypointensity reflects relatively larger myelin content (Ganzetti et al., 2014). Therefore, the T1w to T2w signal intensity ratio should semi-quantitatively reflects the structural white matter integrity, especially after the recently proposed postprocessing algorithm (Ganzetti et al., 2014). This method has been shown to be useful in patients with other neurologic disorders, associated with myelin damage, particularly in multiple sclerosis; also, it is not inferior to the diffusion tensor imaging or magnetization transfer ratio for demyelination measurement (Pareto et al., 2020; Hannoun et al., 2022). The advantages of the T1w/T2w mapping include its simplicity and no increase in the total time of magnetic resonance imaging (MRI) acquisition; also, this method is plausible for retrospective analysis.

On the other hand, myelin damage should result in gray matter loss in adjacent brain regions. It has been shown that there is gray matter atrophy in patients with multiple sclerosis (Eshaghi et al., 2018) or vascular dementia (Li et al., 2017). Fully automatic surface-based MR-morphometry with FreeSurfer is the greatest tool for assessment of the cortical thicknesses and gray matter volumes in different brain areas (Fischl, 2012). There are multiple studies on the usage of this method in patients with different neurologic and psychiatric conditions (Schmaal et al., 2017; Trufanov et al., 2021). Despite that, the data regarding patients with moyamoya angiopathy is limited and controversial, because it is a rare disease. There are only a few published studies with small samples sizes; one of these revealed cerebellar atrophy and precentral cortical atrophy in patients with MMA (Moon et al., 2020), but it was not confirmed in another study (Kazumata et al., 2019).

We hypothesized that chronic ischemia in moyamoya angiopathy leads to loss of myelin integration even in normal-appearing white matter, especially in anterior watershed regions (such as centrum semiovale and middle frontal gyrus), as well as cortical atrophy in adjacent areas, and these changes should correlate with the disease stage. To investigate this hypothesis, we applied the T1w/T2w mapping method and surface-based automatic brain segmentation.

## Materials and methods

### Subjects

The subjects for this study were patients diagnosed with MMA according to the diagnostic guidelines [Guidelines for Diagnosis and Treatment of Moyamoya Disease, 2012], who were hospitalized to the Federal Neurosurgical Center, Novosibirsk from 2014 to 2021. Twenty patients (five males; 36 affected hemispheres; 17–62 years of age; average, 37.8 years), including eight postoperative patients (10 hemispheres), participated in this study (details are provided in Supplementary Table 1). All patients underwent a series of MR imaging scans and a digital subtraction angiography (DSA). During the same period, 17 age- and sex-matched healthy subjects with no history of neurologic or systemic diseases (five males; 34 hemispheres; 16–62 years of age; average, 37.6 years) were evaluated by the same MR imaging protocol. This study was approved by the institutional ethical committee.

### Digital subtraction angiography and disease staging

All DSA procedures were performed with a single C-arm fluoroscopy system (Siemens, Berlin, Germany). A standard angiographic method (with transfemoral approach) and a standard injection protocol for six main arteries of the head were applied in all cases, with a 5F catheter being used to acquire the images. Assessing the severity of the disease, we used the Suzuki staging system, which has been widely applied to evaluate disease progression (Liu et al., 2019). According this classification, Stage I disease characterized by narrowing of the carotid fork, Stage II by the initiation of the moyamoya appearance, Stage III by the intensification of the moyamoya (with disappearance of the middle and anterior cerebral arteries), Stage IV by the minimization of the moyamoya (disappearance of the posterior cerebral artery, and narrowing of individual moyamoya vessels), Stage V by the reduction of the moyamoya (disappearance of all the main cerebral arteries arising from the internal carotid artery system, further minimization of the moyamoya vessels, and an increase in the collateral pathways from the external carotid artery system), and Stage VI by the disappearance of the moyamoya, with cerebral blood flow derived only from the external carotid artery and the vertebrobasilar artery system [Guidelines for Diagnosis and Treatment of Moyamoya Disease, 2012].

### Magnetic resonance imaging acquisition

All MR imaging data were acquired using a 1.5T system (Avanto; Siemens) equipped with an 8-channel receiver head coil. MRI protocol included T1-WI, T2-WI, FLAIR, DWI, MRA, and SWI, and the total acquisition time was approximately 25 min. T1-WI high-resolution sequence (3D VIBE in the axial plane) had the following parameters: TR—6.26 ms, TE—2.42 ms, FOV—256*256 mm, Matrix—256*256, slice thickness—1 mm; T2-WI sequence (TSE in the axial plane) had the following parameters: TR—5000 ms, TE—91 ms, FOV—272*244 mm, Matrix—384*262, slice thickness—5 mm.

### Automatic magnetic resonance- morphometry

Fully automatic surface-based MR-morphometry of high-resolution T1-WI was performed with FreeSurfer v7.2.0 software^1^ and included basic and subcortical segmentation, cortical parcelation, and white matter parcelation. The results were visually checked by the neuroradiologist for each subject and inapplicable areas were excluded from the analysis. Five hemispheres were excluded due to the extended gliotic changes. Additionally, in eight hemispheres, the areas with cystic or gliotic changes were excluded (for detailed information see Supplementary Table 1). Such parameters as regional cortical thickness and white matter regional volumes are used for the following analysis. Individual segmentation results were used to generate binary masks for the centrum semiovale and middle frontal gyrus white matter based on FreeSurfer’s probabilistic atlases (FreeSurferColorLUT).

### T1w/T2w ratio

This step was performed using the MRTool toolbox,^2^ based on the SPM12 software package (Wellcome Department of Imaging Neuroscience, London, United Kingdom^3^). The processing steps for T1w/T2w ratio maps creation included T1-WI and T2-WI co-registration, bias correction, and intensity standardization by the linear scaling procedure, as previously described (Ganzetti et al., 2014). The data of cortical and WM parcelation and subcortical segmentation (from the previous step) was used for binary masks creation in FSL (fslmaths^4^). These binary masks were converted to each subject’s T1w/T2w map’s space by the linear image registration tool (bbregister^5^) implemented in FreeSurfer, according to previously performed automatic segmentation. After that, the binary masks were used as ROIs to extract the T1w/T2w ratio values for each region in FSL (fslstats, see text footnote 4). Supplementary Figure 1 schematically shows the entire MRI postprocessing algorithm.

### Statistical analysis

Statistical analysis was performed using R software.^6^ Regional values from each hemisphere were analyzed independently and unaffected hemispheres were excluded in patients with unilateral disease. Our data was normally distributed. Unpaired *t*-tests were performed to compare the regional values of the T1w/T2w ratio and cortical thickness between the patients with MMA and the healthy controls. Correlation analysis (Pearson correlation test) was performed between regional metrics and the disease stage in patients with MMA. *P* < 0.05 was regarded as statistically significant (without and with FDR correction for three comparisons). The mean values of the studied variables are reported together with ± standard deviation from the mean.

## Results

### Comparison of the regional values of the T1w/T2w ratio and automatic morphometry results between patients and controls

**FIGURE 1:**
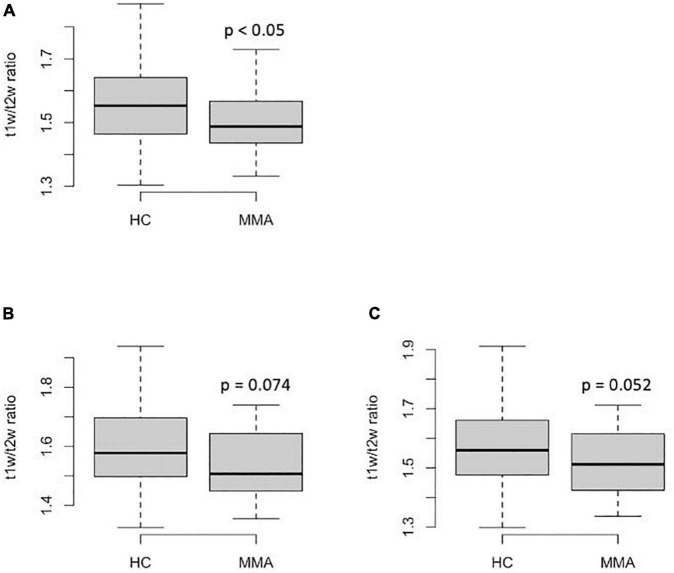
Regional T1w/T2w ratio values from the anterior watershed regions in patients with MMA and healthy controls: **(A)**–centrum semiovale; **(B)**–caudal middle frontal gyrus; **(C)**–rostral middle frontal gyrus. The *P*-values on the graphs are before FDR-correction, adjusted *p* = 0.09 **(A)**, >0.1 **(B)**, and >0.1 **(C)**.

The regional T1w/T2w ratio values from centrum semiovale in patients with MMA were significantly lower than those in healthy controls (*p* < 0.05 before FDR correction and *p* = 0.09 after FDR correction, Figure 1A). The regional T1w/T2w ratio values from middle frontal gyrus white matter (both rostral and caudal portions) tended to be lower in patients than in the controls, but the difference was not statistically significant (*p* < 0.1 and *p* > 0.1 before and after FDR correction, respectively, Figures 1B,C).

**FIGURE 2:**
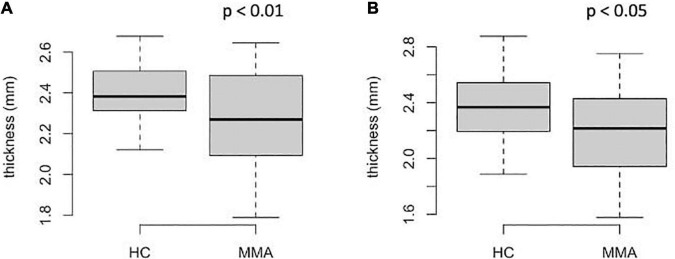
Regional thickness of the middle frontal cortex in patients with MMA and healthy controls: **(A)**–rostral portion; **(B)**–caudal portion. The *P*-values on the graphs are before FDR-correction, adjusted *p* < 0.05 **(A)**, <0.05 **(B)**.

Additionally, the cortical thicknesses from the middle frontal gyrus (both rostral and caudal portions) were significantly lower than those in healthy controls (*p* < 0.05 and *p* < 0.05, before and after FDR correction, Figures 2A,B). The differences in other regional cortical thicknesses and the volumes of subcortical structures were not significant. There were no significant differences in white matter volume of the middle frontal gyrus (both rostral and caudal portions) between groups (*p* > 0.1, not shown). There were no significant differences in such parameters as intracranial volume, total brain volume, and gray matter volume between groups.

Full results are shown in Supplementary Table 2.

### Correlation between the regional values of the T1w/T2w ratio and automatic morphometry results and Suzuki stage

**FIGURE 3:**
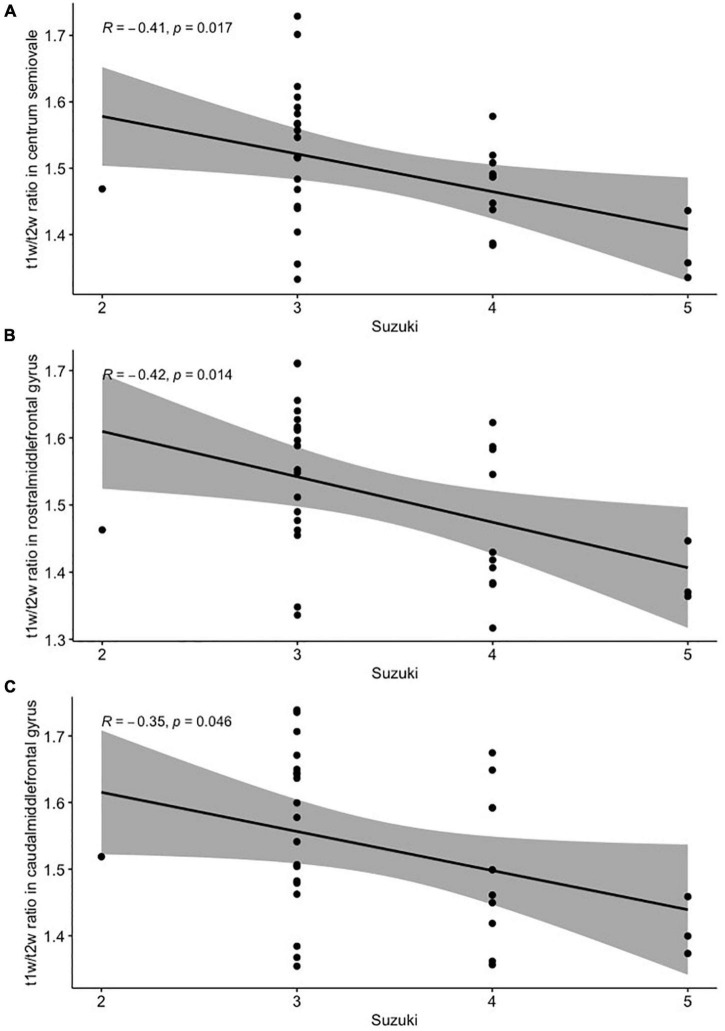
Pearson’s correlation analysis between the Suzuki stage and T1w/T2w ratio from the centrum semiovale **(A)**, the rostral **(B)**, and the caudal **(C)** portions of the middle frontal gyrus white matter.

There were slight but significant negative correlations between the Suzuki stage and the values of the T1w/T2w ratio from the centrum semiovale (*r* = −0.41, *p* < 0.05; Figure 3A) and middle frontal white matter (both rostral and caudal portions; *r* = −0.42, *p* < 0.05 and *r* = −0.35, *p* < 0.05, respectively; Figures 3B,C). For checking the possible impact of hemodynamic improvement after revascularization, we performed additional analysis with assessment of the correlations between Suzuki stage and the values of the T1w/T2w ratio only in patients who didn’t underwent surgery. The correlations were the same (r = −0.41–42, *p* < 0.05 in all three cases, not shown). There were no significant correlations between the regional cortical thicknesses and Suzuki stage (*p* > 0.1, not shown).

## Discussion

Consistent with our hypothesis, statistically significant decreases in regional T1w/T2w ratio values from anterior external watershed regions (centrum semiovale and middle frontal gyrus) were found in patients with MMA as compared to healthy controls. Also, these changes were associated with the decrease of the cortical thickness in the same regions and were negatively correlated with the Suzuki stage. All of this confirms the assumption that watershed regions are the most prone to microstructural changes of the brain parenchyma due to chronic ischemia, especially in the case of steno-occlusive processes.

There are numerous neuroimaging methods for brain white matter integrity assessment, and all of these have some advantages and disadvantages (Buyanova and Arsalidou, 2021). Diffusion tensor imaging with the calculation of fractional anisotropy (FA) and mean, lateral and radial diffusivity (MD, LD, MD) maps is the most popular technique for this goal (Faizy et al., 2020); also, there were attempts of myelinization evaluation based on diffusion-weighted images (Watanabe et al., 2013) with subsequent apparent diffusion coefficient (ADC) calculation. Besides diffusion metrics, there are several neuroimaging approaches for myelination measurement, such as T1-relaxation mapping (Kühne et al., 2021), magnetization transfer ratio and magnetization transfer saturation mapping (Hara et al., 2020), the calculation of the water myelin fraction, or WMF (MacKay and Laule, 2016); and the method of the macromolecular proton fraction assessment, or MPF (Yarnykh, 2016). Various methods have been used in patients with moyamoya angiopathy and revealed the myelin damage, diffuse or focal (Kazumata et al., 2019; Hara et al., 2020; Liu et al., 2020). However, compared to all these methods, T1w/T2w mapping is faster and offers much more simple data acquisition and postprocessing, which is why it is more likely to be used in routine clinical practice. As demonstrated in multiple sclerosis patients, it is not inferior to diffusion tensor imaging or magnetization transfer ratio for measuring demyelination (Pareto et al., 2020; Hannoun et al., 2022). As far as we can tell, it is the first attempt to assess myelin integration in patients with MMA using the T1w/T2w mapping technique.

There is a limited number of papers dedicated to brain atrophy in MMA, which results are controversial. Kazumata et al. (2019) showed the ventricles enlargement, volume reduction of the corpus callosum and basal ganglia, and, more importantly, the prefrontal cortex thinning in patients with MMA compared to the control group. However, these results were not confirmed in the study of Moon et al. (2020); the authors revealed the precentral gyrus gray matter thinning and the cerebellum volume loss. That’s why the middle frontal cortex thinning revealed by our study should be interpreted as preliminary, despite it having high theoretical pathophysiological justification.

The gray matter of the middle frontal gyrus is the part of the prefrontal cortex, and it is known that the prefrontal cortex is associated with cognition, especially with executive functions (Fuster, 2008). Executive functions are cognitive abilities, such as decision making, planning, behavior in a paradigm shift, etc. (Brevers et al., 2015). Unfortunately, because of the retrospective design of our study, it was not possible to assess the cognitive functions of participants. However, previous studies in this field have revealed the cognitive decline in MMA patients and its correlation with structural changes in the brain (Kazumata et al., 2019; Hara et al., 2020).

We revealed the myelin damage in association with gray matter volume loss in the same areas, which demonstrates the complexity of the microstructural changes of the brain parenchyma in chronic ischemic conditions. It has been proposed previously, that myelin damage could be irreversible in MMA (Hara et al., 2020) because it persists even after surgical treatment. On the other hand, an increase in fractional anisotropy values in white matter after revascularization surgery has been shown in another study (Kazumata et al., 2019), which should represent the improvement of the white matter microstructural integrity (remyelination). Interestingly, there was no significant decrease in the middle frontal gyrus white matter volume in our study; it may reflect that myelin damage is potentially reversible. Further investigations with prospective design and more sample sizes are necessary to confirm of this hypothesis.

In this particular study, we decided not to assess the T1w/T2w ratio values from cortical ROIs because there was a relatively low resolution of calculated maps for this goal. But it can be interesting to determine, whether there is “cortical demyelination” in affected regions in patients with MMA, such as, for example, in patients with multiple sclerosis (Granberg et al., 2017) or schizophrenia (Iwatani et al., 2015). Therefore, it may be the subject of further investigations.

This study also has several limitations. First of all, there is a small sample size and participants’ heterogeneity in our study, because MMA is a rare condition. Secondly, our study did not exclude stroke patients, since it is a very common occurrence in the natural course of the disease. Also, some of our patients were previously surgically treated (see Supplementary Table 1), and thus, they had improved hemodynamic conditions. Finally, the retrospective design of our study didn’t allow the cognitive status evaluation for subsequent correlation with revealed gray and white matter changes. Further studies with larger sample sizes are needed to prove our results.

## Conclusion

Using a T1w/T2w mapping method and surface-based MR-morphometry, we found that myelin damage exists in watershed regions in patients with moyamoya angiopathy, in association with cortical atrophy in the same areas, as revealed by MR-morphometry. These changes are correlated with the disease stage. These preliminary results presume that T1w/T2w mapping may be useful for white matter integrity assessment in patients with MMA.

## Data availability statement

The raw data supporting the conclusions of this article will be made available by the authors, without undue reservation.

## Ethics statement

The studies involving human participants were reviewed and approved by Federal Center of Neurosurgery Novosibirsk Local Ethics Committee. Written informed consent to participate in this study was provided by the participants’ legal guardian/next of kin.

## Author contributions

EF, KO, and JR contributed to conception and design of the study. KO organized the database. AS performed the statistical analysis. EF wrote the first draft of the manuscript. KO, AP, RG, DG, AB, and VK wrote sections of the manuscript. All authors contributed to manuscript revision, read, and approved the submitted version.

## Footnotes

1
https://surfer.nmr.mgh.harvard.edu

2
https://www.nitrc.org/projects/mrtool

3
http://www.fil.ion.ucl.ac.uk/spm

4
https://fsl.fmrib.ox.ac.uk/fsl/fslwiki/Fslutils

5
https://surfer.nmr.mgh.harvard.edu/fswiki/bbregister

6
www.r-project.org

## Supplementary material

Supplementary Figure 1 Schematic illustration of the MRI data postprocessing.
