# Supplementary figures and images for: Myelin damage and cortical atrophy in watershed regions in patients with moyamoya angiopathy
Source: Front Neurosci. 2022 Aug 23;16:982829. doi: 10.3389/fnins.2022.982829 (PMC9445365; doi:10.3389/fnins.2022.982829)

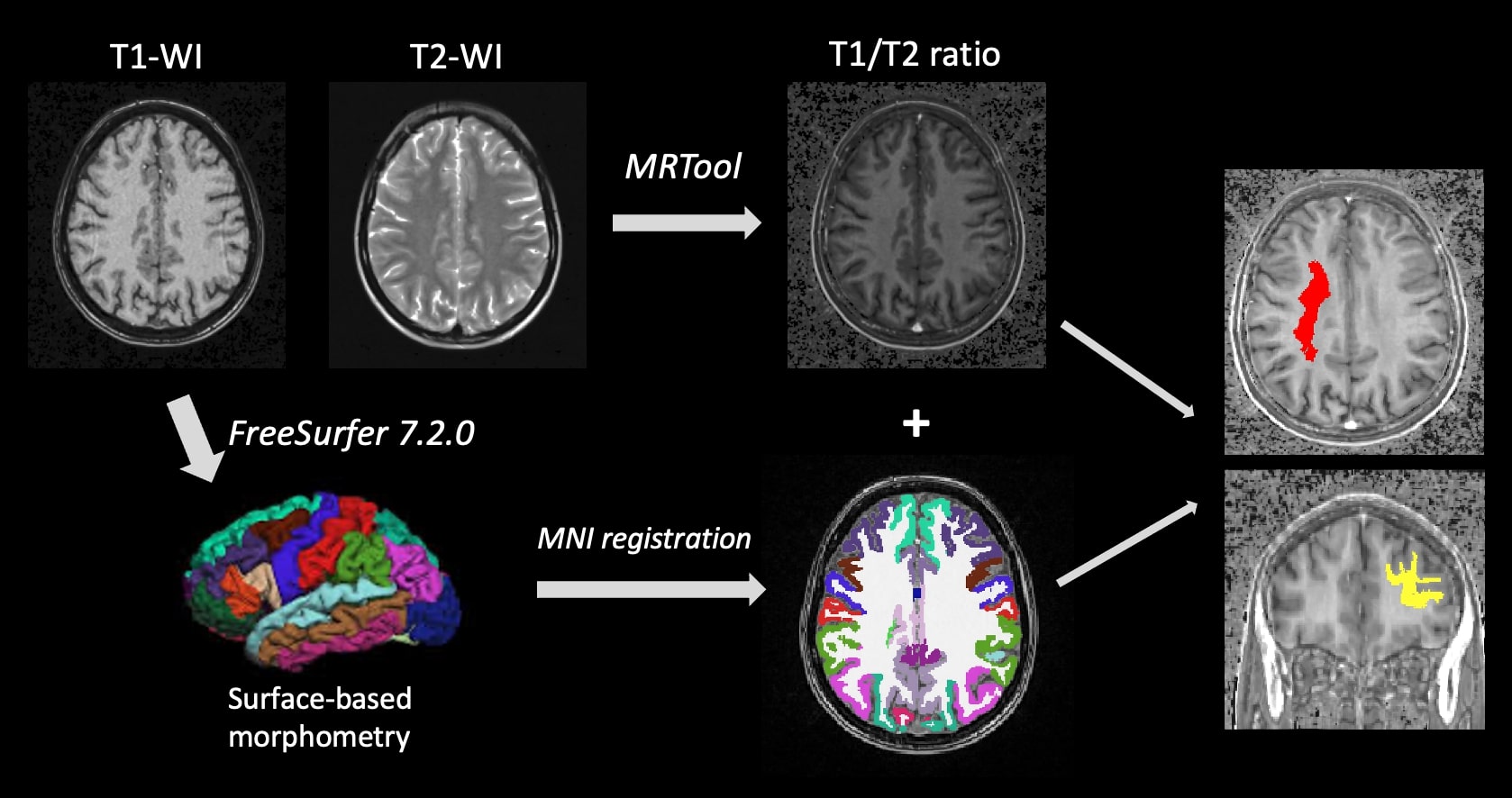

Supplement: Supplementary Figure 1 — Schematic illustration of the MRI data postprocessing. [file Image_1.JPEG]

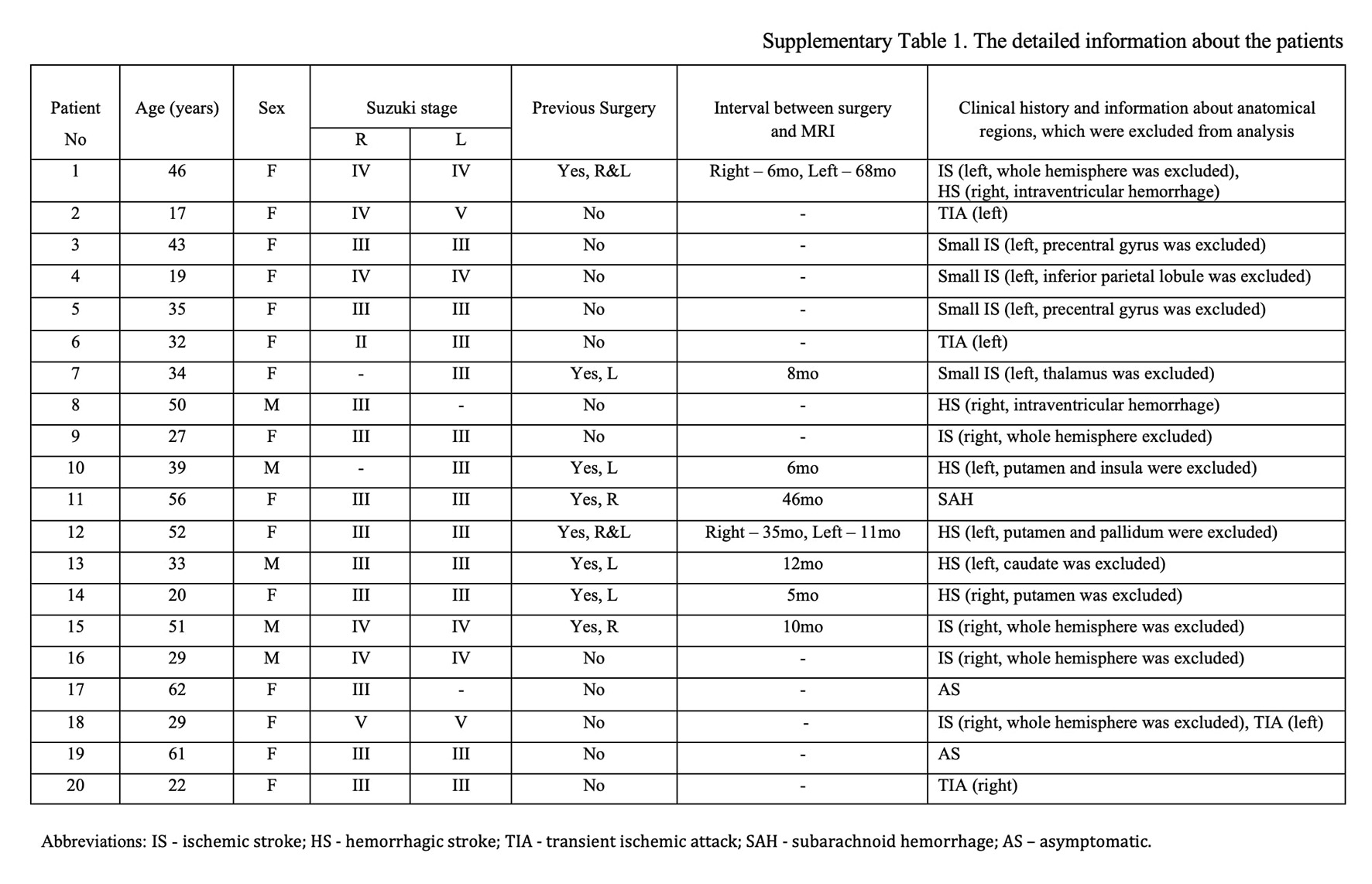

Supplement: Supplementary file 2 [file Image_2.JPEG]

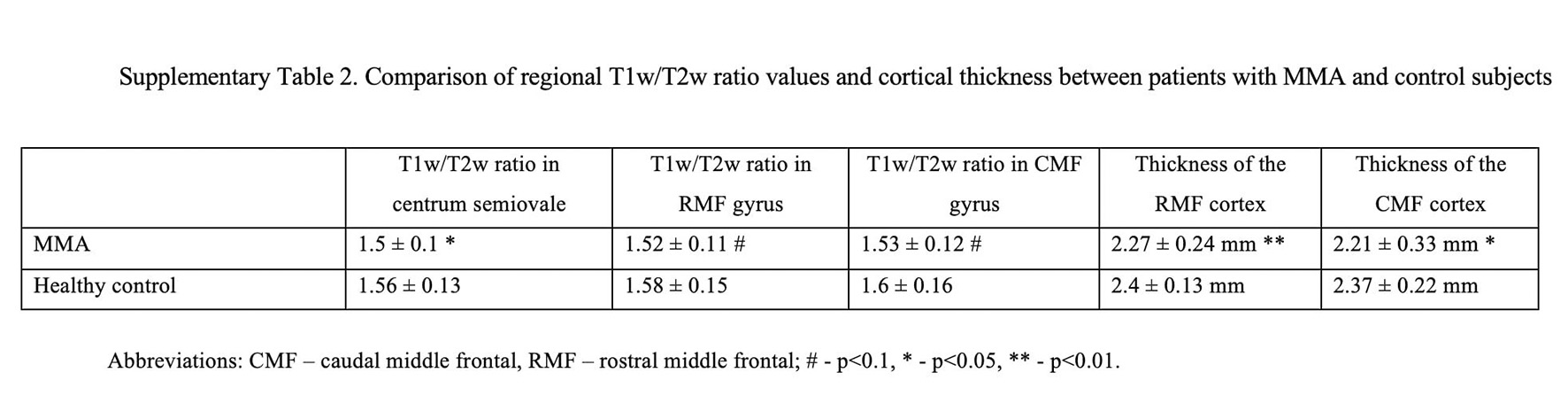

Supplement: Supplementary file 3 [file Image_3.JPEG]
